# The utility of the CADISS® system in the dissection of epidural fibrosis in revision lumbar spine surgery (A case series)

**DOI:** 10.1016/j.amsu.2022.104718

**Published:** 2022-09-22

**Authors:** Marouane Makhchoune, Xavier Collard, Michel Triffaux, Olivier DE Witte, Alphonse Lubansu

**Affiliations:** a Neurosurgery Department, Hospital Center of Wallonie Picarde, Tournai, Belgium; b Department of Spine and Hip Surgery, University Hospital Ambroise Paré, Boulevard Kennedy 2, 7000, Mons, Belgium; c Department of Neurosurgery, Université Libre de Bruxelles, Erasme Hospital, Route de Lennik 808, 1070, Brussels, Belgium

**Keywords:** Spine surgery, CADISS® system, Fibrosis, MESNA, Case series

## Abstract

Spine surgery and spinal fusion surgery are rising. Revision rates following initial surgery are between 8 and 45%. Epidural fibrosis is a common response to spine surgery for most patients and increases complications in revision surgery. Previous research suggests using MESNA (Sodium 2-mercaptoethane sulfonate) in combination with mechanical blunt dissection safely reduces surgical complications. MESNA is a mucolytic agent which selectively cleaves disulphide bonds involved in the adherence and strength of fibrosis, meaning cutting instruments are not needed. The Chemically Assisted DISSection (CADISS®) System is an optimised non-cutting surgical device, consisting of a reconstitution cartridge for MESNA preparation, irrigated surgical instruments, and a footswitch to control MESNA release. This is the first study to investigate the use of the CADISS® System in revision spine surgery.

**Methods:**

This was a prospective, open label, observational case study. We enrolled 21 patients for revision spine surgery with the CADISS® System at two Belgium sites. The primary assessment was the number of successful removals of epidural fibrosis without cutting. The amount of MESNA used, total dissection and procedure time were recorded. For secondary criterion, the surgeons assessed global satisfaction, facilitation of dissection, quickness of action, usability, bleeding reduction and visualisation of the cleavage plane using an 11-point Likert scale (0–10). Due to the exploratory nature, no formal statistical analysis was planned. We calculated the percentage and confidence interval of successful procedures, the medians and corresponding interquartile range of the Likert criterion, and the mean (±SD) of the amount of MESNA used, CADISS® dissection time and total procedure time.

**Results:**

24 fibrosis dissections were performed in 19 patients and 23 were successful (95.8%, CI: 78.9%; 99.9%). The mean amount of MESNA used, mean dissection time and procedure time were 16 ml (±4.94), 16.5 min (±16.1) and 86.3 min (±25.1), respectively. No dural tears were reported. The mean global satisfaction score was 9.0 (8.0–9.0). All other Likert criterion had scores of 8.0 or 9.0, excluding quickness of action, which scored 7.0 (6.0–9.0).

**Conclusions:**

The CADISS® System in revision spine surgery has potential to effectively reduce dissection complications.

## Highlights

• Revision rates following initial surgery are between 8 and 45%. Epidural fibrosis is a common response to spine surgery for most patients and increases complications in revision surgery.
• Previous research suggests using MESNA (Sodium 2-mercaptoethane sulfonate) in combination with mechanical blunt dissection safely reduces surgical complications. MESNA is a mucolytic agent which selectively cleaves disulphide bonds involved in the adherence and strength of fibrosis, meaning cutting instruments are not needed. The Chemically Assisted DISSection (CADISS®) System is an optimised non-cutting surgical device, consisting of a reconstitution cartridge for MESNA preparation, irrigated surgical instruments, and a footswitch to control MESNA release.
• We enrolled 21 patients for revision spine surgery with the CADISS® System at two Belgium sites. The primary assessment was the number of successful removals of epidural fibrosis without cutting. The CADISS® System in revision spine surgery has potential to effectively reduce dissection complications.

## 1 Introduction

Spine surgery is on the rise globally [[1], [2], [3]]. For instance, the annual incidence in Norway increased by 54% between 1999 and 2013 to 119.9 per 100,000 people, of which 14.8% were reoperations [4]. Spinal fusion surgery is also on the rise, evident from studies in the US and Europe [[5], [6], [7], [8], [9], [10]]. The annual rate increased 4.33 times from 1998 to 2011 [11] and revision rates following primary spinal fusion surgery range from 8 to 45% [12]. Understanding data from health services regarding surgery and its complications and burden is crucial for improving and planning future services and methods [4]. Epidural fibrosis is a common response to spine surgery and is identified in the majority of patients, one study reports severe fibrosis affected 83.3% of patients [13]. Revision surgery is more complicated than primary surgery due to the development of fibrosis and scar tissues from the first operation [14]. Fibrotic tissues are difficult to dissect and may result in incidental durotomy (ID), or dural tears, and other intraoperative complications. The prevalence of ID in revision spine surgeries is typically between 7 and 17% [14,15]. Therefore, a system for aiding dissections in revision surgery would be helpful for reducing potential complications. This is the first study to investigate the use of The Chemically Assisted DISSection (CADISS®) System in revision spine surgery.

**Fig. 1:**
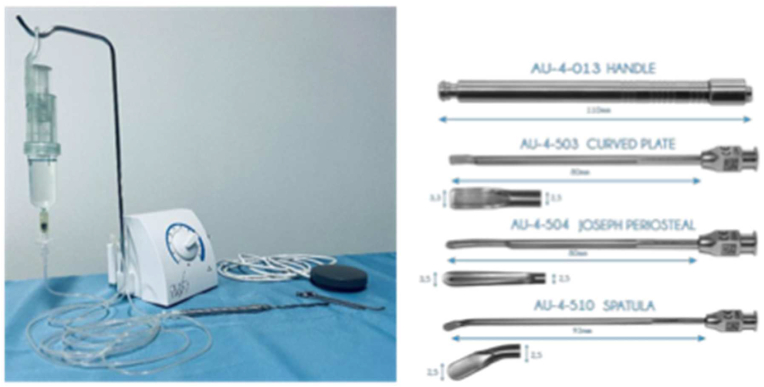
The CADISS® Remote System and non-cutting instruments used in dissections.

The CADISS® System uses a topical formulation of MESNA (Sodium 2-mercaptoethane sulfonate) to facilitate dissection of pathological and fibrotic tissues. MESNA is a mucolytic agent which selectively cleaves disulphide bonds responsible for the adherence and strength of the fibrosis, meaning cutting instruments are not necessary [16]. Previous evidence indicates using MESNA in this way is a safe and useful method for supporting surgery, in orthopaedic, ear, nose and throat and gynaecological fields [[16], [17], [18], [19], [20]]. Evidence suggests it reduces surgery side effects and relapses, and makes the surgical procedure quicker and significantly easier [15,16]. A double blinded clinical trial of 30 patients suggests using this method for revision lumbar spine surgery reduces complications, as well surgical time and difficulty [15]. In this trial, MESNA solution in a syringe was connected to an irrigated non-cutting mechanical instrument. This has been optimised into the CADISS® device, which was CE Marked for musculoskeletal surgery in 2019. The CADISS® device consists of several elements (see Fig. 1); a single use reconstitution cartridge for preparing the MESNA solution (a 30 ml solution with a 5% concentration), irrigated surgical instruments, and a footswitch to control dispensing the solution. In addition, the composition of the solution was optimised for surgical application. While the 30-patient trial applied the solution using a syringe, these additions applied creating the CADISS® device allow for more controlled release of MESNA compared with the previous method. The reconstitution of CADISS® is straightforward and the device was designed to eliminate particle risk. Therefore, the risk of accidental contamination for the patient may be reduced. Further trials using MESNA for chemically assisted dissection surgery have been encouraged [16]. Therefore, this study aims to meet the need for investigation using the CADISS® System for revision spine surgery and to verify previous results of using MESNA solution and its associated benefits in regular practice for both patients and surgeons.

## 2 Materiels and methods

This was a prospective, open label observational case study. We enrolled 21 patients for surgery with the CADISS® System at two Belgium sites. Two patients discontinued before surgery. The primary efficacy endpoint was the number of dissections of epidural fibrosis effectively completed without cutting. For the secondary efficacy measurements, an 11-point Likert scale (0–10) was used. For example, “The topical application is easy to control” or “Bleeding is reduced”. The score was defined in comparison with current practice, with a score of 5 indicating no difference to current practice and a score of 10 indicating full satisfaction of the criterion. The two surgeons involved in the study have extensive experience of the same dissection without the CADISS® System, and therefore, were able to make informed comparisons when assessing performance with the Likert Scale. We recorded the amount of MESNA used, the total CADISS® dissection time and the total time to complete the surgical procedure. To assess the safety of the procedure, each investigator collected adverse events (AEs) throughout the observation period, performed a physical examination; an electrocardiogram; and collected vital signs at baseline and during the surgery day.

Due to the observational and exploratory nature of this study, no formal sample size calculation was performed, and no formal statistical analysis was planned. We calculated the percentage of successful procedures and the corresponding confidence interval (CI), the medians and the corresponding interquartile range (IQR) of the Likert scale scores for each criterion and the mean (±SD) of the amount of MESNA used, the CADISS® dissection time and the total procedure time.

We conducted the trial in accordance with Good Clinical Practice guidelines and the Declaration of Helsinki and obtained approval from the independent ethics committees of the two sites (Ethic Committees University Hospitals Erasme and Ambroise Paré). Participants signed written informed consents before study entry. The trial was funded by AuXin Surgery, Belgium.

## 3 Results

To reflect current surgical practice, the inclusion criteria were intentionally wide: ≥18 years old, >30 kg, and eligible for spine revision surgery at least one year after primary surgery. Patients were excluded if they had hypersensitivity to MESNA; were pregnant, breastfeeding or had a pregnancy wish; had participated in a study involving an investigational drug or device in the past three months; or were under tutorship or trusteeship. The 19 participants who underwent surgery had a mean height of 169.0 cm (SD: 8.43) and a mean weight of 77.89 kg (SD: 22.58) with a mean BMI of 27.01 kg/m2 (SD: 6.43) [Table 1]. The mean amount of MESNA solution used during surgery was 16 ml (±4.94), out of the full 30 ml. The mean dissection time was 16.5 min (±16.1), and the mean procedure time was 86.3 min (±25.1).

**Table 1:** Demographics of patients who underwent revision spine surgery with the CADISS® device.

	Height (cm)	Weight (kg)	BMI (kg/m2)
Mean (N = 19)	169	77.89	27.01
SD	8.43	22.58	6.43

N = number of patients; SD = standard deviation.

In total 24 dissections of fibrosis were performed in 19 patients. Of these, 23 were successful (95.8%, CI: 78.9%; 99.9%). The surrounding tissue involved in the dissection process were neurinoma L4/L5 (n = 1), nerve root (n = 1), dura matter [14], lumbar spine (n = 2), narrow lumbar canal (n = 1), material (n = 4) and bone (n = 1) [Table 2].

**Table 2:** Surrounding tissue involved in the dissections.

Surrounding tissue	Number of dissections	Number of successful dissections
Neurinoma L4/L5	1	0
Nerve root	1	1
Dura matter	14	14
Lumbar spine	2	2
Narrow lumbar canal	1	1
Material	4	4
Bone	1	1

Mean scores for the secondary efficacy measurements on the Likert scale are presented in Table 3. The median global satisfaction score was 9.0 (IQR: 8.0–9.0), with no difference between sites. Median scores for facilitation of dissection, bleeding reduction, and visualisation of the cleavage plane were 9.0 (IQR: 8.0–10.0), 9.0 (IQR: 7.0–9.0), and 8.0 (IQR: 8.0–9.0), respectively. The median score for the quickness of action was 7 (IQR: 6.0–9.0). Useability, for both the ease of using the remote kit and control of the topical application, had median scores of 9.0 (IQR: 8.0–10.0).

**Table 3:** Likert scale results for secondary endpoints for using the CADISS® device in revision spine surgery.

Secondary Endpoint	Likert Score mean (IQR)	IQR
Global Satisfaction Score (n = 19)	9.0	8.0–9.0
Topical application reveals cleavage plane (n = 19)	8.0	8.0–9.0
Quickness of action (n = 18)	7.0	6.0–9.0
Detachment of fibrosis is facilitated (n = 19)	9.0	8.0–10.0
Bleeding is reduced (n = 16)	9.0	7.0–9.0
The remote kit is easy to use (n = 19)	9.0	8.0–10.0
The topical application is easy to control (n = 19)	9.0	8.0–10.0

n = number of observations; IQR = interquartile range.

The two patients who did not undergo surgery, discontinued due to a “patient decision”, rather than in relation to the CADISS® system. No dural tears were reported for any of the 19 patients who underwent surgery. In total 32 AEs occurred in 15 patients. Of these, 7 (in 5 patients) were assessed as serious (dyspnea, surgical site infection, sciatica, pain in thigh, lumbar pain, infected seroma, and seroma drainage). One serious AE (SAE, surgical site infection) was assessed as related to the use of the CADISS® system. All AEs resolved without sequelae.

Two individual case studies of patients in this trial were of interest. One 46-year-old patient with a large history of lumbar spine surgery had her first surgery in March 2015 for L4L5 TLIF lumbar arthrodesis and had a surgery with the CADISS® system in August 2020. However, her underwent various other surgical procedures in between. After her initial surgery, there were complications of a deep hematoma superinfection & multi-sensitive *Escherichia coli* which required two debridings. However, the patient still felt significant lumbar pain afterwards and required another surgery in June 2016 for L4L5 arthrodesis. After this operation, her pain improved significantly but one year later in 2017 the lumbar pain returned. This pain continued even with physiotherapy and facet infiltrations. A computerised tomography (CT) scan, and confirmed by magnetic resonance imaging (MRI), showed the L4L5 arthrodesis was good but canal narrowing of the L3L4 discopathy was worsening. Revision surgery using the CADISS® system allowed for an easy removal of the fibrosis at both the posterior arthrodesis material level and at the dural sac. There was no dural breach in this operation.

Another case concerns a 65-year-old patient who had had one previous surgery for L3L5 canal recalibration in May 2019. After this operation, a disabling of the right L5 sciatica occurred, which did not improve following conservative treatment. Then, an MRI revealed two cysts, one a large arthosynovial cyst impinging on right L5, and the other, a small L3L4 Dr cyst. In June 2020, a reoperation on the initial surgical site used the CADISS® system and therefore the fibrosis was easily able to be detached without affecting the dural sac. Both cysts were removed and there were no post-operative complications.

This case series has been reported in line with the PROCESS Guideline [27].

## 4 Discussion

**Fig. 2:**
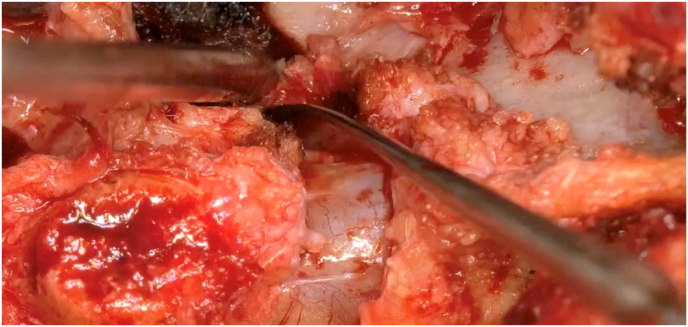
Easy dissection of fibrosis in revision spine surgery with The CADISS®.

The results indicate the CADISS® system may be an effective method for the dissection of fibrosis in revision spine surgery and could be quicker and easier compared to current practice (Fig. 2). The 95.8% of successful dissections over all the operations and high satisfaction and usability scores suggest CADISS® may have advantages that could benefit both surgeons' practice and patients’ surgery and post-surgery experience. However, there were some limitations to the study and understanding these will allow the indicated conclusions in this study to be further investigated in the future.

Surgery was only unsuccessful in one patient. It involved dissection of a neurinoma L4/L5 tissue. This unsuccessful dissection was the first patient to be operated on by this surgeon and the contact time between the MESNA solution and fibrosis seemed to be shorter. In addition, the surgeon may still have been unfamiliar with using the CADISS® system. This explains the lower score for global satisfaction that was observed, which is due to the low outlying score for this one operation. All other following dissections were successful, and all dissections performed by the other surgeon were also successful. As only one SAE was thought to be related to the use of CADISS®, it suggests the risk of additional complications would be low with this method.

Quickness of action was the only endpoint with a lower median score, with 7 on the Likert scale. This can be explained by the waiting time of around 5 min that is required for the chemical action of the MESNA to take effect on the fibrotic tissues [21]. Although the score is lower than other endpoints, it is still above 5, which indicates an improvement of the speed of dissection in comparison to current practice, according to the surgeons’ subjective opinion. Therefore, although a lower score was observed here, it still suggests the CADISS® system may be a quicker method than the typical method in use for these kinds of spine surgery procedures.

Dural tears are one of the most common complications of spine surgery, particularly in the lumbar area and for revision surgeries [14,[22], [23], [24], [25]]. As previously mentioned, dural tear rates are usually between 7 and 17% for lumbar revision spine operations [14]. Furthermore, dural tears sustained with posterior lumbar spinal decompression and/or fusion surgery can significantly increase the length of hospital stay, risk of readmission and the overall 90-day hospital cost [26]. They have also been found to increase the risk of venothromboembolic events by 1.46 times and meningitis by 6 times, both of which can lead to patient morbidity [26]. In the current study, no dural tears were observed meaning there was a complete absence of ID when using the CADISS® method. Considering the typical incidence of ID and associated risks, the absence seen here supports the suggestion CADISS® is a safe method for this kind of surgery and may even reduce normal rates of usually observed complications. This is also made apparent when looking at the individual case studies we reported, particularly the 46-year-old patient who had experienced multiple lumbar spine surgeries. This patient had numerous complications and continuing back pain after previous operations, but she had no intra or post-operative complications, including dural tears, after the operation with CADISS® up to hospital discharge. This is reflected in the case of the second patient we discussed as well. Although these are only two cases which have been discussed within the full study of 24 operations, the results are promising when considering typical dural tear rates.

The results are also in line with the conclusions of a previous study, discussed in our background, in which a chemically assisted mechanical dissection with MESNA was used [15]. In comparison to this previous study using MESNA to chemically assist dissection in revision lumbar spine surgery, there were both differences and similarities. The previous study included a blinded control group, however, as already mentioned, it only used chemically assisted dissection. The current study used the CADISS® method, still using MESNA application but with a solution composition developed for surgical application and a more sophisticated operational system in a typical practice setting. The remote kit and foot pedal of the CADISS® system allowed the surgeons to decide more precisely the volume of MESNA that was appropriate to use for each operation and gave them more control over the release of MESNA. In the 2010 study, less dural tears (1/15) were observed in the MESNA-controlled compared with their control group (4/15), while none were observed in the current study. Alongside the high global satisfaction and usability scores in this study, this suggests CADISS may have an advantage.

There were a few limitations with the current study. Primarily, the small cohort size and the absence of a direct comparison mean concrete conclusions regarding the benefits of CADISS®-assisted dissection cannot yet be made. Further clinical studies using a larger cohort and a comparison group, using the CADISS® device in regular practice, are necessary to fully verify the advantages using of this system. As it stands, there is some potential for CADISS® System to improve current practices and it ought to be investigated further.

If further investigations are made, and the preliminary conclusions from this study are confirmed, it could be greatly beneficial to this surgical field. If the CADISS® method proves to be advantageous, it could improve surgical practice for surgeons and reduce costs to health services. In addition, patients undergoing spine surgery may be able to have a smoother operation and lower risks of complications and pain post-surgery. Therefore, further research into the CADISS® system for revision spine surgery, and in other surgical fields, is encouraged.

## 5 Conclusion

The results suggest the CADISS® System has potential for aiding dissection of fibrosis in revision lumbar spine surgery. All surgeries were successful, excluding the first patient, which was likely due to the shorter contact time with the MESNA solution. Surgeon satisfaction and useability of the system were rated highly, indicating this method could be advantageous over conventional methods. The CADISS® System

Has potential to improve current practices of fibrosis removal during revision spine surgery, however, further clinical research will be needed to confirm this conclusion.

## Ethical approval

Written informed consent for publication of their clinical details and/or clinical images was obtained from the patient, Ethical approval has been exempted by our institution.

## Sources of funding

None.

## Author contributions

Marouane MAKHCHOUNE: Corresponding author and writing the paper, Xavier COLLARD: writing the paper, Michel TRIFFAUX: writing the paper, Olivier DE WITTE: writing the paper, Alphonse LUBANSU: Correcting the paper.

## Declaration of competing interest

The authors declare having no conflicts of interest for this article.

## Research registration unique identifying number (UIN)

None.

## Trial registry number – ISRCTN

ClinicalTrials.gov Identifier: NCT05016739 - first registration: 23/08/2021 https://clinicaltrials.gov/ct2/show/NCT05016739.

## Guarantor

MAKHCHOUNE MAROUANE.

## Provenance and peer review

Not commissioned, externally peer reviewed.
